# Effects of vasopressinergic receptor agonists on sublingual microcirculation in norepinephrine-dependent septic shock

**DOI:** 10.1186/cc10453

**Published:** 2011-09-19

**Authors:** Andrea Morelli, Abele Donati, Christian Ertmer, Sebastian Rehberg, Tim Kampmeier, Alessandra Orecchioni, Alessandro Di Russo, Annalia D'Egidio, Giovanni Landoni, Maria Rita Lombrano, Laura Botticelli, Agnese Valentini, Alberto Zangrillo, Paolo Pietropaoli, Martin Westphal

**Affiliations:** 1 Department of Anesthesiology and Intensive Care, University of Rome, "La Sapienza," Viale del Policlinico 155, Rome I-00161, Italy; 2 Department of Neuroscience-Anesthesia and Intensive Care Unit, Università Politecnica delle Marche, Via Tronto 10, Torrette di Ancona I-60020, Italy; 3 Department of Anesthesiology and Intensive Care, University Hospital of Muenster, Albert-Schweitzer-Strasse 33, Muenster D-48149, Germany; 4 Department of Anesthesia and Intensive Care, Università Vita-Salute San Raffaele, Via Olgettina 60, Milan I-20132, Italy

## Abstract

**Introduction:**

The present study was designed to determine the effects of continuously infused norepinephrine (NE) plus (1) terlipressin (TP) or (2) arginine vasopressin (AVP) or (3) placebo on sublingual microcirculation in septic shock patients. The primary study end point was a difference of ≥ 20% in the microvascular flow index of small vessels among groups.

**Methods:**

The design of the study was a prospective, randomized, double-blind clinical trial. NE was titrated to maintain mean arterial pressure (MAP) between 65 and 75 mmHg after establishment of normovolemia in 60 septic shock patients. Thereafter patients (*n *= 20 per group) were randomized to receive continuous infusions of either TP (1 μg/kg/hour), AVP (0.04 U/minute) or placebo (isotonic saline). In all groups, open-label NE was adjusted to maintain MAP within threshold values if needed. The sublingual microcirculatory blood flow of small vessels was assessed by sidestream dark-field imaging. All measurements, including data from right heart catheterization and norepinephrine requirements, were obtained at baseline and 6 hours after randomization.

**Results:**

TP and AVP decreased NE requirements at the end of the 6-hour study period. The data are medians (25th and 75th interquartile ranges (IQRs)): 0.57 μg/kg/minute (0.29 to 1.04) vs. 0.16 μg/kg/minute (0.03 to 0.37) for TP and 0.40 μg/kg/minute (0.20 to 1.05) vs. 0.23 μg/kg/minute (0.03 to 0.77) for AVP, with statistical significance of *P *< 0.05 vs. baseline and vs. placebo. There were no differences in sublingual microcirculatory variables, systemic hemodynamics, oxygen transport and acid-base homeostasis among the three study groups during the entire observation period. The proportions of perfused vessels increased in relation to baseline within all study groups, and there were no significant differences between groups. The specific data were as follows (median (IQR)): 9.7% (2.6 to 19.8) for TP, 8.9% (0.0 to 17.8) for AVP, and 6.9% (3.5 to 10.1) for placebo (*P *< 0.05 vs. baseline for each comparison), as well as perfused vessel density 18.6% (8.6 to 36.9) for TP, 20.2% (-3.0 to 37.2) for AVP, and 11.4% (-3.0 to 19.4) for placebo (*P *< 0.05 vs. baseline for each comparison).

**Conclusions:**

The present study suggests that to achieve a MAP of 65 to 75 mmHg in septic patients treated with NE, the addition of continuously infused low-dose TP or AVP does not affect sublingual microcirculatory blood flow. In addition, our results suggest that microcirculatory flow abnormalities are mainly related to other factors (for example, volume status, timing, hemodynamics and progression of the disease) rather than to the vasopressor *per se*.

**Trial registration:**

ClinicalTrial.gov NCT00995839

## Introduction

The current guidelines on the management of patients with septic shock recommend the use of vasopressor agents, such as norepinephrine (NE) or dopamine, to increase peripheral vascular resistance and preserve organ perfusion following adequate volume therapy [1]. Since microvascular dysfunction plays a crucial role in the pathophysiology of septic shock and organ dysfunction [2,3], special attention should be paid to the effects of vasopressor agents on tissue perfusion and microcirculatory blood flow [4,5]. In this regard, recent studies have demonstrated that the administration of NE to achieve a target MAP between 60 and 90 mmHg does not worsen microcirculatory perfusion [6,7]. Whether the vasopressor *per se*, rather than other factors (for example, volume status, timing, hemodynamics and progression of the disease), plays a role in preserving or deteriorating capillary perfusion in septic shock is still not fully understood.

Despite their efficacy in reducing catecholamine requirements and stabilizing hemodynamics in patients with vasodilatory shock, there are concerns that the addition of vasopressinergic agents may further impair microcirculation because of their pronounced vasoconstrictive potency. Although V_1 _receptor agonism may cause excessive vasoconstriction, concomitant stimulation of vasodilatory V_2 _receptors may potentially improve microcirculatory blood flow. Whether the reduction of catecholamine requirements following hemodynamic support with vasopressors has a positive effect on the microcirculation is likewise unknown. Despite these relevant open questions, the available data on the effects of vasopressinergic agonists on the microcirculation in human septic shock are scarce and remain to be determined.

The objective of the present randomized, controlled, double-blind clinical study was therefore to compare, for a predefined goal MAP in patients with septic shock treated with NE, the effects of adding continuously infused low-dose terlipressin (TP), arginine vasopressin (AVP) or placebo on microcirculatory perfusion as judged by modifications of sublingual microvascular blood flow using sidestream dark-field (SDF) imaging [8].

## Materials and methods

### Patients

After approval by the local Institutional Ethics Committee, the present study was performed in an 18-bed multidisciplinary ICU at the Department of Anesthesiology and Intensive Care of the University of Rome "La Sapienza." Informed consent was obtained from the patients' next of kin. Enrollment of patients started in November 2008 and ended in March 2010. We enrolled patients who fulfilled the criteria of septic shock [1] and required NE to maintain MAP ≥ 65 mmHg despite appropriate volume resuscitation (pulmonary arterial occlusion pressure (PAOP) = 12 to 18 mmHg and right atrial pressure (RAP) = 8 to 12 mmHg) [1].

Exclusion criteria were: age < 18 years, pronounced cardiac dysfunction (that is, cardiac index (CI) ≤ 2.2 L/minute/m^2 ^in the presence of PAOP > 18 mmHg), severe liver dysfunction, significant valvular heart disease, present coronary artery disease, pregnancy, present or suspected acute mesenteric ischemia or vasospastic diathesis (for example, Raynaud's syndrome or related diseases).

All patients underwent lung-protective mechanical ventilation using a volume-controlled mode, which was adjusted to maintain plateau < 30 cmH_2_O [1]. In all patients, positive end-expiratory pressure was set at a level ranging from 7 to 15 cmH_2_O. The ventilatory settings remained unchanged throughout the study period. All patients were appropriately analgo-sedated using sufentanil and midazolam and received intravenous hydrocortisone (300 mg/day) as a continuous infusion. Activated protein C was administered at the discretion of the attending physician.

### Measurements

Systemic hemodynamic monitoring of the patients included the use of a pulmonary artery catheter (7.5-French; Edwards Lifesciences, Irvine, CA, USA) and a radial artery catheter. MAP, RAP, mean pulmonary arterial pressure (MPAP) and PAOP were measured at end-expiration. Heart rate was analyzed by continuous recording of an electrocardiogram with ST segments monitored. CI was measured using the continuous thermodilution technique (Vigilance II; Edwards Lifesciences). Arterial and mixed venous blood samples were taken to measure oxygen tension and saturation as well as carbon dioxide tension, standard bicarbonate and base excess (BE). Mixed venous oxygen saturation (SvO_2_) was measured discontinuously by intermittent mixed venous blood gas analyses.

### Microvascular network

Microvascular blood flow was visualized by means of a SDF imaging device (MicroScan; MicroVision Medical, Amsterdam, The Netherlands) equipped with a 5× magnification lens [8]. The optical probe was applied to the sublingual mucosa after gentle removal of saliva with a gauze swab. Three discrete fields were captured with caution to minimize motion artefacts. Individual sequences of approximately 15 seconds were analyzed offline with the aid of dedicated software (Automated Vascular Analysis 3.0 software; Academic Medical Center, University of Amsterdam, Amsterdam, The Netherlands) in a randomized fashion by a single investigator who was unaware of the study protocol. The "De Backer Score" was calculated as described previously [8]. It is based on the principle that the density of the vessels is proportional to the number of vessels crossing arbitrary lines. In this score, three equidistant horizontal and three equidistant vertical lines are drawn on the screen. The De Backer Score can be calculated as the number of the small, medium and large vessels crossing the lines divided by the total length of the lines [8]. Vessel density was calculated as the total vessel lengths divided by the total area of the image [8]. Both indices were automatically calculated by the utilized software. Perfusion was then categorized by eye as present (normal continuous flow for ≥ 15 seconds), sluggish (decreased but continuous flow for ≥ 15 seconds), absent (no flow for ≥ 50% of time) or intermittent (no flow for < 50% of time) [8]. The proportion of perfused vessels (PPVs) was calculated as follows:

100×(total number of vessels−[no flow+intermittent flow])/total number of vessels

Perfused vessel density (PVD) was calculated by multiplying vessel density by the PPVs [8]. Vessel size was determined with the aid of a micrometer scale. Small vessels were defined as vessels with a diameter < 20 μm. Since our investigation was primarily focused on small vessels, calculations were separately performed for vessels with a diameter smaller than 20 μm. Microvascular flow index of small vessels (MFIs) was used to quantify microvascular blood flow in these vessels. Therefore, flow was characterized as absent 0, intermittent 1, sluggish 2, or normal 3 [8]. For each patient, values obtained from the three mucosa fields were averaged. To assess flow heterogeneity between the different areas investigated, we used the heterogeneity index. The latter was calculated as the highest site flow velocity minus the lowest site flow velocity, divided by the mean flow velocity of all sublingual sites [8]. Percentage changes from baseline for all variables were calculated as follows [9]:

$$\text{dVariable}=100\times \left[\left(\text{Valu}{\text{e}}_{\text{6 hours}}∕\text{Valu}{\text{e}}_{\text{BL}}\right)-1\right]$$

### Study design

After having established normovolemia (PAOP = 12 to 18 mmHg and CVP = 8 to 12 mmHg) [1] and a MAP ≥ 65 mmHg using NE, patients were randomized to one of three study groups. Whereas patients allocated to the TP group received a continuous TP infusion of 1 μg/kg/hour, patients in the AVP group were treated with a continuous infusion of AVP of 0.04 U/minute. The control group received a continuous infusion of isotonic saline as placebo. All the investigated drugs were administered in a blinded fashion. In all three groups, open-label NE was titrated to maintain goal MAP between 65 and 75 mmHg if necessary. Fluid challenge (6% hydroxyethylstarch 130/0.4) was performed to maintain PAOP and CVP at baseline ± 3 mmHg during the 6-hour study period.

Systemic hemodynamic variables, microcirculatory flow variables, blood gases and NE requirements were determined at baseline and 6 hours after randomization. After the 6-hour intervention period, study drugs were discontinued, and in all three groups open-label NE was titrated to maintain MAP between 65 and 75 mmHg.

### Statistical analyses

The primary end point of the present study was the difference in MFIs between groups after 6 hours of treatment. An *a priori *analysis of sample size revealed that 20 patients per group were required to demonstrate a minimum difference in means of 20% between groups for the primary end point with an assumed standard deviation of 20%, a test power of 80% and an α error of 5%. The above-mentioned assumptions were based on prior experience with the respective methodology [10].

Data are expressed as medians (25th and 75th percentiles) if not otherwise specified. SigmaStat 3.10 software (Systat Software, Inc., Chicago, IL, USA) was used for statistical analysis. Baseline and demographic data were compared with an analysis of variance (ANOVA) on ranks or a χ^2 ^test as appropriate. Differences in microvascular and hemodynamic variables between groups were analyzed by ANOVA on ranks. Time-dependent changes within each group were determined with a signed-rank test. *P *< 0.05 was considered statistically significant for all tests.

## Results

### Demographic data

**Figure 1:**
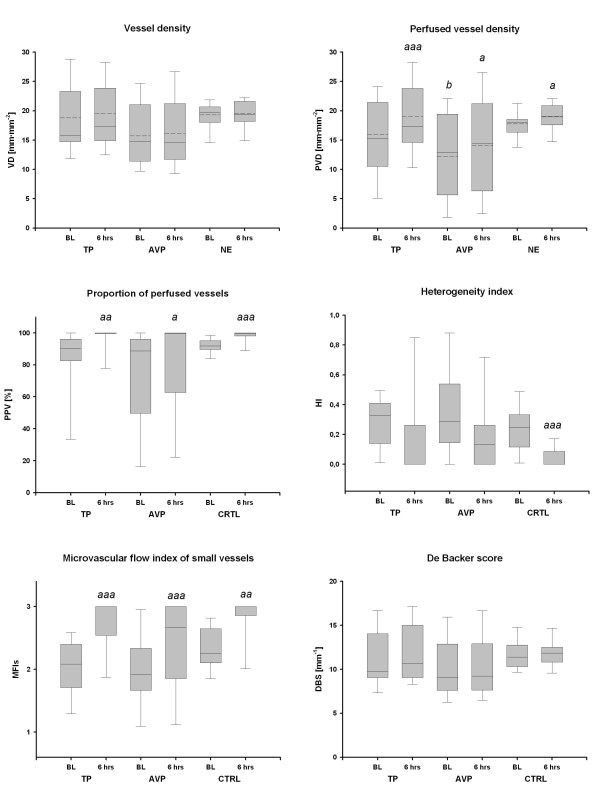
**Absolute changes in microcirculatory variables**. AVP, arginine vasopressin; BL, baseline; DBS, De Backer Score; HI, heterogenity index; MFI, microvascular flow index of small vessels (Ø < 20 μm); NE, norepinephrine; CTRL, control; PPV, proportion of perfused vessels; PVD, perfused vessel density; TP, terlipressin; VD, vessel density. Solid lines, median; dashed lines, mean; boxes, 25th and 75th percentiles; whisker caps, 10th and 90th percentiles. ^a^*P *< 0.05, ^aa^*P *< 0.01, ^aaa^*P *< 0.001 vs. BL; ^b^*P *< 0.05 vs. CTRL.

Baseline characteristics of the study patients, including age, gender, Simplified Acute Physiology Score II, origin of septic shock and time from onset of septic shock until study drug infusion were similar among groups (Table 1). In addition, there were no significant differences between groups at baseline in any of the investigated hemodynamic, metabolic or microcirculatory variables, except for lower PVD in the AVP group than in the control group (Figure 1). Activated C protein was administered in eight patients in the NE group and in six patients in both TP and AVP groups.

**Table 1:** Characteristics of the study patients

Characteristics	Terlipressin (*n *= 20)	Arginine vasopressin (*n *= 20)	Control (*n *= 20)	*P *value
Age, years	65 (51 to 71)	71 (48 to 78)	66 (58 to 75)	*P *= 0.31
Male gender (%)	55%	70%	60%	*P *= 0.61
SAPS II	50 (46 to 59)	53 (49 to 58)	54 (46 to 59)	*P *= 0.83
Cause of septic shock (*n*)				*P *= 0.50
Cholangitis		1		
Meningitis	1	3		
Necrotizing fasciitis	1	2	2	
Peritonitis	6	5	5	
Pancreatitis		1		
Pneumonia	12	8	13	
Onset of septic shock, hours*	37 (21 to 44)	35 (27 to 45)	34 (22 to 52)	*P *= 0.89

SAPS II, Simplified Acute Physiology Score II. Data are medians (25th and 75th percentiles) unless otherwise indicated. *Onset of septic shock defines the time elapsed from the onset of septic shock until administration of study drug.

### Hemodynamic and oxygen transport variables and norepinephrine requirements

**Figure 2:**
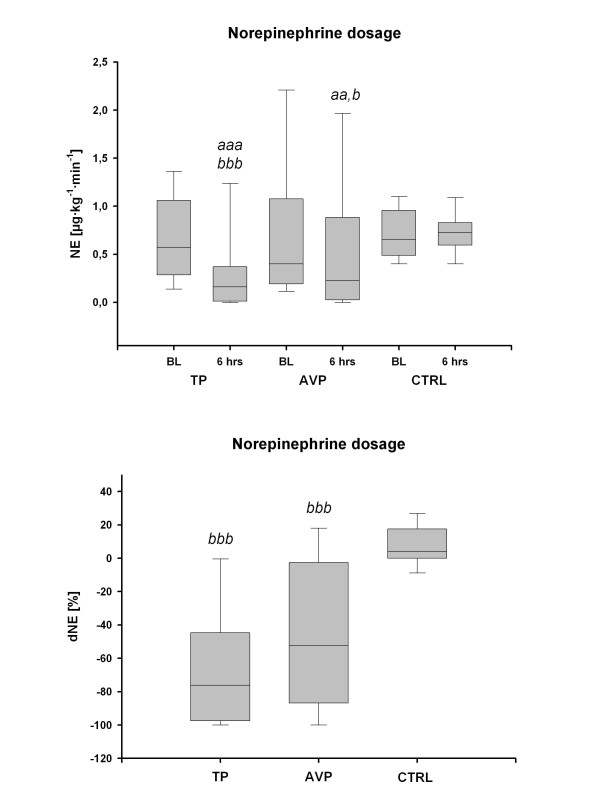
**Norepinephrine requirements**. The upper panel shows norepinephrine (NE) infusion rates in the terlipressin (TP), arginine vasopressin (AVP) and control (CTRL) groups at baseline (BL) and at 6 hours. The lower panel shows relative NE changes (dNE) after 6 hours compared to baseline in each study group. Solid lines, median; dashed lines, mean; boxes, 25th and 75th percentiles; whisker caps, 10th and 90th percentiles. ^aa^*P *< 0.01, ^aaa^*P *< 0.001 vs. BL; ^b^*P *< 0.05, ^bbb^*P *< 0.001 vs. controls.

Systemic and pulmonary hemodynamics as well as acid-base variables and fluid input are given in Table 2. There were no significant differences among groups, except for a lower hemoglobin concentration in the TP group than in the control group at 6 hours. NE requirements were markedly reduced after 6 hours in the TP group (0.57 μg/kg/minute (0.29 and 1.04) vs. 0.16 μg/kg/minute (0.03 to 0.37); with each *P *< 0.001 vs. controls and baseline values) and in the AVP group (0.40 μg/kg/minute (0.20 and 1.05) vs. 0.23 μg/kg/minute (0.03 and 0.77); with each *P *< 0.05 vs. controls and baseline values) as compared to both baseline and the control group (0.66 μg/kg/minute (0.51 to 0.92) vs. 0.73 μg/kg/minute (0.63 to 0.83); *P *= 0.11 vs. baseline) (Figure 2).

**Table 2:** Hemodynamic and metabolic data of the study patients

Parameters	TP (*n *= 20)	AVP (*n *= 20)	Control (*n *= 20)	*P *value
CI (L/min/m^2^)				
Baseline	3.8 (3.1 to 5.4)	4.0 (3.2 to 4.9)	4.0 (3.5 to 4.6)	0.96
6 hours	4.0 (3.1 to 5.1)	3.5 (3.0 to 4.0)*	4.0 (3.4 to 4.9)	0.28
HR (bpm)				
Baseline	104 (86 to 113)	99 (83 to 119)	104 (86 to 111)	1.00
6 hours	88 (82 to 100)	91 (75 to 117)	91 (85 to 114)	0.64
MAP (mmHg)				
Baseline	71 (68 to 75)	72 (69 to 75)	71 (68 to 75)	0.97
6 hours	74 (72 to 75)	72 (68 to 75)	74 (68 to 75)	0.31
MPAP (mmHg)				
Baseline	30 (27 to 35)	31 (28 to 36)	29 (27 to 32)	0.42
6 hours	29 (24 to 33)	29 (26 to 34)	31 (26 to 33)	0.65
PAOP (mmHg)				
Baseline	16 (14 to 19)	18 (16 to 20)	18 (15 to 20)	0.26
6 hours	17 (13 to 20)	18 (15 to 22)	18 (14 to 20)	0.45
RAP (mmHg)				
Baseline	13 (10 to 15)	15 (12 to 17)	13 (10 to 14)	0.13
6 hours	13 (10 to 15)	14 (11 to 16)	11 (10 to 14)	0.22
LVSWI (g/m/m^-2^)				
Baseline	29 (24 to 41)	26 (24 to 35)	30 (22 to 37)	0.85
6 hours	32 (26 to 44)	24 (23 to 39)	30 (21 to 44)	0.11
DO_2_I (mL·min^-1^/m^2^)				
Baseline	437 (391 to 604)	460 (396 to 677)	470 (399 to 550)	0.92
6 hours	461 (364 to 587)	416 (369 to 473)	509 (431 to 600)	0.13
O_2_-ER (%)				
Baseline	25 (23 to 30)	26 (22 to 30)	31 (27 to 36)	0.05
6 hours	32 (25 to 35)	29 (24 to 38)	35 (29 to 41)	0.15
S_v_O_2 _(%)				
Baseline	75 (68 to 77)	73 (67 to 80)	69 (66 to 76)	0.30
6 hours	70 (66 to 77)	70 (59 to 77)	67 (59 to 72)	0.26
Hb_a _(g/dL)				
Baseline	8.3 (8.0 to 9.5)	8.9 (8.3 to 9.8)	8.7 (8.0 to 9.3)	0.50
6 hours	8.3 (8.0 to 8.8)*	8.6 (8.1 to 9.7)	9.0 (8.6 to 10.0)	0.005
pH_a _(-log_10_c(H^+^))				
Baseline	7.30 (7.24 to 7.34)	7.31 (7.27 to 7.37)	7.31 (7.28 to 7.36)	0.62
6 hours	7.39 (7.31 to 7.43)	7.33 (7.28 to 7.40)	7.31 (7.25 to 7.37)	0.08
BE (mmol/L)				
Baseline	-3.9 (-7.0 to -1.2)	-3.1 (-8.0 to 1.8)	-2.9 (-5.9 to 1.6)	0.65
6 hours	-2.2 (-4.7 to 2.4)	-3.4 (-5.7 to 1.5)	-4.0 (-6.2 to 0.5)	0.41
Lactate (mmol/L)				
Baseline	1.8 (1.2 to 2.9)	2.3 (1.4 to 3.6)	2.5 (1.9 to 3.0)	0.67
6 hours	2.1 (1.5 to 2.8)	2.3 (1.4 to 3.6)	2.6 (2.1 to 3.7)	0.45
Fluid input (mL/6 hours)				
Baseline	n/a	n/a	n/a	n/a
6 hours	875 (790 to 950)	910 (830 to 975)	895 (800 to 990)	0.51

AVP, arginine vasopressin; BE, base excess; CI, cardiac index; DO_2_I, systemic oxygen delivery index; Hb_a_, arterial hemoglobin concentration; HR, heart rate; LVSWI, left ventricular stroke work index; MAP, mean arterial pressure; MPAP, mean pulmonary arterial pressure; O_2_-ER, oxygen extraction ratio; PAOP, pulmonary arterial occlusion pressure; pH_a_, arterial *Potentia hydrogenii*; RAP, right atrial pressure; S_v_O_2_, mixed venous oxygen saturation; TP, terlipressin; VO_2_I, systemic oxygen consumption index. Data are medians (25th and 75th percentiles). *P < 0.05 vs. control.

### Microcirculatory variables

**Figure 3:**
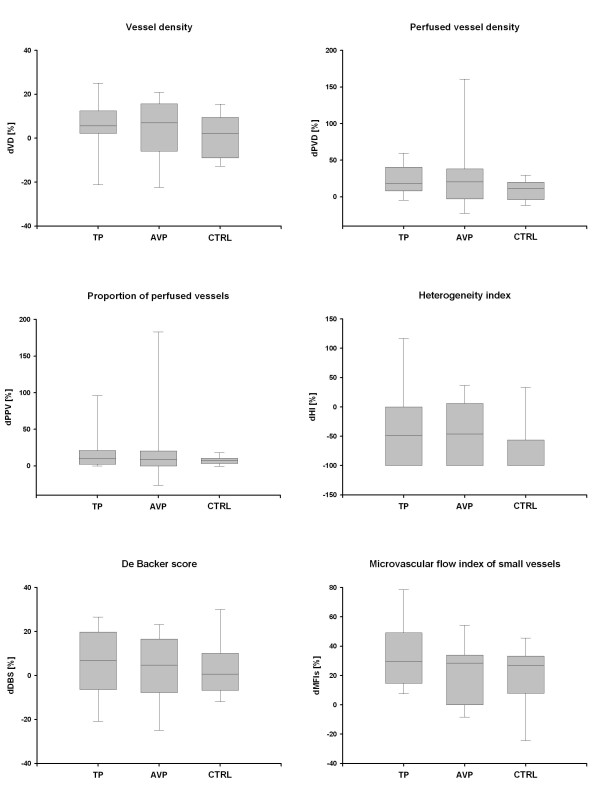
**Relative changes in microcirculatory variables**. Data represent relative changes from baseline (BL) at 6 hours. AVP, arginine vasopressin; dDBS, relative changes in De Backer Score; dHI, relative changes in heterogeneity index; dMFI, relative changes in microvascular flow index of small vessels (Ø < 20 μm); dPPV, relative changes in proportion of perfused vessels; dPVD, relative changes in perfused vessel density; dVD, relative changes in vessel density; CTRL, control; TP, terlipressin. Solid lines, median; dashed lines, mean; boxes, 25th and 75th percentiles; whisker caps, 10th and 90th percentiles.

Absolute and relative changes (compared to baseline) of microcirculatory variables of the three study groups are presented in Figures 1 and 3. PVD, PPV and MFIs significantly increased in all groups (each *P *< 0.05 vs. baseline). The heterogeneity index tended to decrease in all groups, but this change was significant only in the control group. None of the absolute or relative changes in microcirculatory variables was significantly different among the three study groups.

## Discussion

The major finding of the present study is that in fluid-resuscitated septic shock patients treated with NE to maintain a MAP between 65 and 75 mmHg, the addition of continuously infused low-dose TP and AVP does not affect sublingual microcirculatory blood flow compared with placebo. Although TP and AVP were effective in reducing NE requirements, there were no clinically relevant effects on the microcirculation.

The current guidelines for the treatment of septic shock recommend the use of vasopressor agents to achieve a MAP ≥ 65 mmHg [1]. While pressure-guided resuscitation is usually effective in restoring MAP, microcirculatory blood flow may not be linearly improved [4,5,11]. This assumption is supported by studies demonstrating that although the administration of NE to achieve incremental targets for MAP between 60 and 90 mmHg does not negatively affect the microcirculation, it does not correct preexisting microcirculatory flow abnormalities [6,7]. Conversely, in the presence of normovolemia, an improvement of microcirculatory perfusion can theoretically be obtained by vasodilator agents, because it dilates afferent arterioles while reducing efferent venous pressure [11,12]. From a physiological perspective, AVP and (to a lesser extent) TP may exert beneficial effects on the microcirculation compared to NE, because they also exert some vasodilatory effects by nitric oxide (NO) release secondary to V_2 _receptor stimulation [13-15].

Despite the different actions of these agents on V_1a _and V_2 _receptors, the present study failed to demonstrate differences between the combinational therapies with TP plus NE, AVP plus NE and sole NE infusion in terms of microcirculatory variables. This finding may imply either that TP and AVP have similar intrinsic activity on V_1a _and V_2 _receptors or that V_2_-mediated vasorelaxant effects [13-15] are attenuated and not hemodynamically relevant. The latter assumption is supported by the fact that the study drugs were administered in the setting of progressed septic shock, where V_2_-mediated vasodilation may be impaired as a result of overproduction of NO. On the other hand, the tendency toward lower NE requirements in the TP group may be explained by different intrinsic activities of the respective AVP and TP concentrations at the V_1a _receptor site.

Recently, the safety of supplemental AVP has been demonstrated in the Vasopressin and Septic Shock Trial (VASST), with a potential survival benefit in patients with less severe septic shock that may support administration of the drug in the early phase of the disease [16]. Moreover, experimental studies [17,18] and clinical studies [19] have provided evidence of a superiority of first-line TP over AVP or sole NE in stabilizing cardiovascular hemodynamics in septic shock. In addition, it has been reported that selective V_2 _receptor antagonism rather than V_2 _receptor stimulation stabilized cardiopulmonary hemodynamics while attenuating metabolic acidosis and tissue injury, thereby limiting organ dysfunction in early experimental septic shock [20]. A review of the results of the present study and the available literature appears to show that there are not only advantages of selective [21] or relatively selective V_1a _receptor agonists, such as TP over mixed vasopressinergic agonists like AVP, but also, more importantly, that the relationship between vasopressinergic receptor agonists and the timing of administration (early vs. late) may be crucial in preserving or deteriorating organ function.

In line with the above-referenced studies [16-21], and supported by the results of our present study, it appears that delayed administration of TP or AVP may not translate into microcirculatory advantages over sole NE, although TP and AVP are still effective in reducing catecholamine requirements. The right timing of administering vasopressinergic agents, however, is still debated. In this context, Kampmeier *et al. *[22] recently reported that early TP infusion reduced catecholamine and fluid requirements compared with delayed TP therapy and placebo in ovine septic shock.

In harmony with the effects on the microcirculation, and in line with previous experimental and clinical studies [17-19,23], we did not notice differences in arterial pH or in lactate concentrations following TP or AVP administration, suggesting a lack of drug-related impairment of cellular oxygenation as well as the absence of differences in resuscitation quality between groups. In this context, it is also important to note that the multicenter VASST study [16] demonstrated no differences between AVP and sole NE in the rate of overall adverse events.

Interestingly, we observed a time-dependent improvement in some microcirculatory variables in all study groups. The beneficial evolution in the NE group over time is in agreement with a recent study by Boerma *et al. *[24], who reported an increase in MFIs even in the placebo group. In this regard, it may be possible that some patients experienced a beneficial evolution with respect to their course of disease during the study period. Another possible explanation is that fluid therapy administered over a short observational period, such as in our protocol, resulted in further recruitment of the microcirculation in some of the patients [25,26]. However, it is important to note that only limited amounts of fluids were given (see Table 2) and that changes were similar between groups.

In the present study, we did not find differences between the study drugs when vasopressor support was titrated to maintain MAP between 65 and 75 mmHg. Nonetheless, we cannot exclude the possibility that a lower threshold MAP would have generated different results, thereby further reducing excessive NE requirements. It also cannot be excluded that the involvement of inflammatory mechanisms mediated by leukocyte activation and cytokine release, as well as hemorheological factors related to the progression of the disease, affected the microcirculatory blood flow more than the choice of vasopressor. Evaluation of these factors, however, was beyond the scope of the present study.

Our study has some limitations that we must acknowledge. First, our protocol did not allow us to draw conclusions regarding whether the observed findings were related mainly to a direct effect of vasopressinergic agents on microcirculation or to a concomitant reduction of NE dosage *per se*. However, we chose this protocol to mimic the clinical scenario, in which AVP and TP are currently used as adjuvant vasopressor support in established, NE-dependent septic shock. Since there are no equivalent doses of AVP and TP for continuous infusion, we decided to evaluate the effects of fixed doses of the study drugs on microcirculatory blood flow while maintaining the threshold MAP with titrated NE infusion. In this regard, it might be argued that a weight-adjusted TP dose was compared with a fixed AVP dose, and thus the chosen doses might not have been pharmacologically equivalent. Therefore, it is possible that the TP dose was relatively higher than the AVP dose. Second, we chose changes in MFIs as the primary end point of this study. Since we investigated only a small number of septic shock patients treated over a brief period, the risk of false-negative or false-positive results in a study with numerous microcirculatory variables has to be taken into account. Moreover, the marginally significant difference in PVD at baseline (*P *= 0.04) represents a limitation of the present study. However, the fact that > 20 variables were compared with a significance threshold of 5% indicates a high probability of significant differences in the absence of true clinical relevance. Since the difference in PVD was the only one observed at baseline, it probably had no major impact on the quality of the present data. However, it cannot be definitively excluded that the opposite is the case. In addition, we cannot rule out the possibility of divergent microcirculatory effects in response to more prolonged administration of the study drugs. When looking at the technique adopted in the present study, we have to acknowledge that whereas SDF imaging allows real-time imaging of the intact microcirculation in the clinical setting, the assessment of some microcirculatory variables that result from this technique remain semiquantitative and the data reliability may be affected by level of technical expertise and interobserver bias. Furthermore, we investigated the changes in microvascular perfusion of the sublingual mucosa, which may not necessarily be representative of alterations in other tissues [2,27,28]. Whether applying a different method to determine fluid responsiveness would have generated different results cannot be answered by the present study.

## Conclusions

In summary, this study is the first to show that in patients with fluid-resuscitated septic shock treated with NE to maintain MAP between 65 and 75 mmHg, the addition of TP, AVP or placebo has similar effects on the sublingual microcirculation. At the investigated doses, the addition of TP and AVP reduced NE requirements without changing sublingual microvascular blood flow. The results of the present study suggest that microcirculatory flow abnormalities are mainly related to other factors (for example, volume status, timing, hemodynamics and progression of the disease) rather than to the vasopressor *per se*.

## Key messages

• In septic patients treated with NE to achieve a MAP of 65 to 75 mmHg, the addition of continuously infused TP or AVP does not affect sublingual microcirculatory blood flow.

• The potential advantages of TP or AVP over sole NE with regard to microcirculation might be limited to the early phases of septic shock.

• Microcirculatory flow abnormalities are related mainly to other factors (for example, volume status, timing, hemodynamics and progression of disease) rather than to the vasopressors *per se*.

## Abbreviations

BE: arterial base excess; CI: cardiac index; dHI: relative changes of heterogeneity index; dMFI: relative increases of microvascular flow index of small vessel; dMFIm: relative increases of microvascular flow index of medium vessel; DO_2_I: systemic oxygen delivery index; dPVD: relative increase in perfused vessel density; HI: heterogeneity index; HR: heart rate; LVSWI: left ventricular stroke work index; MAP: mean arterial pressure; MFIm: microvascular flow index of medium vessel; MFI: microvascular flow index of small vessel; NE: norepinephrine; O_2_-ER: oxygen extraction rate; PAOP: pulmonary arterial occlusion pressure; PPV: proportion of perfused vessels; PVD: perfused vessel density; RAP: right atrial pressure; RVSWI: right ventricular stroke work index; S_a_O_2_: arterial oxygen saturation; SAPS II: Simplified Acute Physiology Score II; S_v_O_2_: mixed venous oxygen saturation.

## Competing interests

The authors declare that they have no competing interests.

## Authors' contributions

AM, CE and MW planned the study, were responsible for its design and coordination and drafted the manuscript. AZ and GL participated in the study design and helped to draft the manuscript. SR and TK participated in the design of the study, performed the statistical analysis and helped to draft the manuscript. AD, AO, ADE, ADR, MRL and AV analyzed the SDF images and helped to draft the manuscript. PP participated in the study design, helped to draft the manuscript and obtained funding. AM, CE, AD and MW revised the manuscript.
